# Study of the Serum Copper Levels in Patients with Major Depressive Disorder

**DOI:** 10.1007/s12011-016-0720-5

**Published:** 2016-05-05

**Authors:** Krzysztof Styczeń, Magdalena Sowa-Kućma, Marcin Siwek, Dominika Dudek, Witold Reczyński, Paulina Misztak, Bernadeta Szewczyk, Roman Topór-Mądry, Włodzimierz Opoka, Gabriel Nowak

**Affiliations:** 1 Department of Affective Disorders, Chair of Psychiatry, Jagiellonian University Medical College, Kraków, Poland; 2 Department of Neurobiology, Laboratory of Trace Elements Neurobiology, Institute of Pharmacology PAS, Kraków, Poland; 3 Department of Analytical Chemistry, University of Science and Technology, Kraków, Poland; 4 Department of Pharmacobiology, Jagiellonian University Medical College, Kraków, Poland; 5 Department of Epidemiology and Population Studies, Institute of Public Health, Jagiellonian University Medical College, Kraków, Poland; 6 Department of Inorganic and Analytical Chemistry, Jagiellonian University Medical College, Kraków, Poland

**Keywords:** Copper, Depression, Biomarkers, Affective disorders, Unipolar depression, MDD

## Abstract

Copper may be involved in the pathophysiology of depression. Clinical data on this issue are very limited and not conclusive. The purpose of the study was to determine the copper concentration in the serum of patients with major depressive disorder and to discuss its potential clinical usefulness as a biomarker of the disease. A case–control clinical study included 69 patients with current depressive episode, 45 patients in remission and 50 healthy volunteers. Cu concentration was measured by electrothermal atomic absorption spectrometry (ETAAS). The mean serum copper level in depressed patients was slightly lower (by 11 %; not statistically significant) than in the control group. Furthermore, there was no significant difference in Cu^2+^ concentration between depressive episode and remission, nor between remission and control group. In the remission group were observed significant correlations between copper levels and the average number of relapses over the past years or time of remission. There was no correlation between serum copper and severity of depression, as measured by HDRS and MADRS. The obtained results showed no significant differences between the copper concentration in the blood serum of patients (both with current depressive episode and in remission) and healthy volunteers, as well as the lack of correlations between the copper level in the active stage of the disease and clinical features of the population. Our study is the first conducted on such a large population of patients, so the results may be particularly important and reliable source of knowledge about the potential role of copper in depression.

## Introduction

Copper (Cu) is the third most abundant trace metal (behind iron and zinc) present in every tissue and is required for essential body functions. As a cofactor for numerous enzymes, it plays an important role in the biochemical processes, including erythropoiesis, cellular respiration, peptide amidation, iron, cholesterol and glucose metabolism, pigment formation, and hormones biosynthesis (see [1] for review). These metal ions are also necessary for the proper development and functioning of the central nervous system; low copper level may result in incomplete development, while excess concentration maybe injurious [2]. Among the body organs, the brain is the one of the most copper-rich (next to the heart and liver) [3].

The total human body contains only 75–100 mg, and the recommended daily dosage for adult men and women is 0.9 mg/day (1–1.3 mg/day for pregnant or lactating women). Copper is relatively a stable component of the blood, and its concentration usually remains between 100 and 130 mg/100 ml of the serum [4, 5]. Approximately 90 % of the copper in the blood is incorporated into ceruloplasmin, which is responsible for carrying copper to tissues that need the mineral [6, 7]. Proper absorption and metabolism of copper requires an appropriate balance with the minerals zinc and manganese. Because zinc can compete with copper in the small intestine and interfere with its absorption, persons who supplement with inappropriately high levels of zinc and lower levels of copper may increase their risk of copper deficiency [8]. That is why the plasma/serum ratio of copper to zinc (optimal 0.70–1.00) is clinically more important than the blood concentration of either of these trace metals. A crucial role in maintaining proper cellular zinc and copper concentration play metallothioneins, which have the capacity to bind both these ions through the thiol group of its cysteine residues. In the human body, large quantities are synthesized primarily in the liver and kidneys and their production is dependent on availability of the dietary minerals [8].

Copper homeostasis is of great importance to human health. Its imbalance is implicated directly or indirectly in the pathogenesis of numerous disorders. Deficit of Cu (rather rare and often co-exist with other nutritional deficiencies) may affect connective tissue leading to vascular and skeletal problems, causes neuronal degeneration, anemia, and cardiac and immune dysfunctions. Copper deficiency is associated with the following diseases: aceruloplasminemia (very low or absence of ceruloplasmin), Huppke–Brendl syndrome, familial amyotrophic lateral sclerosis, Huntington’s disease, Menkes disease, and occipital horn syndrome [9, 10]. In turn, excess amounts of copper may result in cellular instability and damage due to the oxidative potential of the free metal [5]. Copper toxicity symptoms include nausea, vomiting, abdominal pain and cramps, headache, dizziness, weakness, and diarrhea. High Cu concentration is observed in Alzheimer’s, Parkinson’s, and Wilson’s diseases and may also lead to decline in intelligence in young adolescents [8, 9]. Limited data also suggests the potential importance of copper in the development of neuropsychiatric disorders, including depression [6, 11–16], and several compelling arguments can confirm this fact.

Firstly, copper is an essential component of some enzymes (monoamine oxidase—MAO, dopamine β-hydroxylaze—DBH, and tyrosine hydroxylase) involved in the proper turnover of catecholamines, which disturbances can lead to the development of depression [17, 18]. Secondly, copper concentration and even more balance between its reduced (Cu^+^) and oxidized form (Cu^2+^) plays a fundamental role in the oxidative and nitrosative stress (O&NS) processes, which may be one of the main cause of mood disorders (e.g., major depressive disorder—MDD) [4, 8, 19, 20]. Furthermore, Cu is required for structural and catalytic properties of some antioxidant enzymes (e.g., lysyl oxidase and superoxidase dismutase), which are important free radical scavengers and prevent oxidative damages of cells [5]. Many studies also suggest that induction of O&NS pathways in depression is accompanied by activation of the inflammatory response and acute phase (AP) proteins [21]. Thus, elevated serum concentration of ceruloplasmin (a key protein involved in the storage of copper), a widely recognized AP protein, in depressed patients may be a further evidence of the potential role of Cu ions in the course of depression [22–24].

Copper concentration in the extracellular space is generally low (0.2–1.7 μM), but during the neurotransmission in some parts of the brain, it can raise even dozens of times. However, the elevated levels of these ions may have important biological significance. In particular, released of copper ions may inhibit *N*-methyl-*D*-aspartate (NMDA) and α-amino-3-hydroxy-5-methyl-4-isoxazolepropionic acid (AMPA) receptor function and thereby protect neurons from glutamatergic excitotoxity [25–28]. The disturbances in glutamatergic transmission are the basis of glutamate hypothesis of depression [29].

All the above data confirm the involvement of copper in the regulation of important biological processes, which may be critical in the development of mood disorders. So far, very little is known about alterations in Cu concentration in depression. There are only few studies on this topic. Thus, we decided to measure the copper level in the blood serum of a relatively large population of patients with MDD (depending on the current stage of the disease) and to discuss its potential clinical usefulness as a biomarker of the disease.

## Methods

This study was a part of a large research project entitled “De-Me-Ter” (depression–mechanisms–therapy), the aim of which was to examine the relationship between the symptomatology of affective disorders and the serum concentrations of some biometals, inflammatory, and oxidative stress markers, potentially involved in the pathophysiology of these diseases [30].

### Recruitment of the Study Participants

The study participants were recruited among the in- and outpatients of the Department of Psychiatry, University Hospital (Jagiellonian University Medical College, Cracow, Poland) in the period from 21 September 2006 to 30 July 2013.

The people meeting the DSM-IV-TR criteria for major depressive disorder (MDD) (both in active phase and in remission) and healthy volunteers were included in the study. All participants signed the informed consent forms and were provided with detailed information (both verbally and in writing) about the aims and characteristics of the study. Each potential study participant had the opportunity to ask questions about the study before signing the consent and all those questions were answered by the doctor responsible for the recruitment. The Jagiellonian University Bioethics Committee have approved this study (decision number KBET/77/B/2009; 25.06.2009).

The exclusion criteria (selected on the basis of precise history taking) were described in detail in our previous report [30], but the main were as follows: lack of signed consent; diagnosis of a severe psychiatric disorder other than MDD (for example: schizophrenia, schizoaffective disorder, bipolar disorder); addictive diseases (excluding addiction to nicotine and caffeine); diagnosis of a severe personality disorder; presence of severe somatic disorders (acute or chronic); breastfeeding; or pregnancy. During the study, all the patients were receiving pharmacotherapy with proven efficacy (mono- or polytherapy; for more details, see [30]).

The control group was recruited through advertisements on hospital notice boards, by referral from hospital staff, or their relatives and friends. This group consisted of individuals with no present and past history of severe and chronic somatic or psychiatric diseases, without history of substance use disorders (except for caffeine and nicotine abuse), and with no psychiatric disorders in the first-degree relatives.

### The Diagnostic Tools

The severity of depressive symptoms was measured with the Montgomery–Asberg Depression Rating Scale—MADRS [31] and the Hamilton Rating Scale for Depression – HDRS [32].

### Collection and Processing of Blood Samples and Quantitative Analysis of Copper in Blood Serum Samples

According to the study protocol, from each patient and healthy volunteer no more than 9.8 ml of blood was obtained from a brachial vein using the Monovette system. After the cloth formation the blood samples were centrifuged for 30 min at 1800 RPM. The obtained serum samples were stored at −80 °C until use.

After thawing, the serum copper levels were measured by a electrothermal atomic absorption spectrometry—ET AAS. The authors used Perkin Elmer spectometer Model 3110 (USA) equipped with a Perkin Elmer HGA-600 graphite furnace. The following measurements’ conditions were used: pretreatment temperature was set to 950 °C and atomization to 2300 °C; the wavelength was set at 324.8 nm and slit to 0.7 nm. The samples were diluted appropriately to fit into the linear range (10–50 ng/ml) of calibration curves. Standards (Copper standard solution; Merck Millipore Corp., Darmstadt, Germany) and samples were prepared as water solutions. Diluted samples were homogenized by means of sonification. Despite dilution, no sample pretreatment procedures were applied prior to quantitative elements determination. All the measurements were performed in triplicate. The accuracy was tested by means of recovery analysis, which for Cu was in the range of 96–103 %.

### Statistical Methods

The *χ* ^2^ test was used in order to analyze the differences between the qualitative variables. The Shapiro–Wilk test was performed in order to evaluate the normal distribution of quantitative data. Because of the absence of the normal distribution of data, we used the Kruskal–Wallis ANOVA or the Mann–Whitney *U* test. Correlations between quantitative variables—due to lack of normal distribution—were analyzed with the Spearman’s rank correlation.

## Results

One hundred and fourteen patients (86 women and 28 men) who met the DSM-IV-TR criteria for MDD (69 patients with depressive episodes and 45 in remission) and 50 healthy people (36 women and 14 men) were recruited for the case–control study. All of the patients were treated according to current guidelines using the following drugs: selective serotonin reuptake inhibitors (SSRI), serotonin–norepinephrine selective inhibitors (SNRI), tricyclic antidepressants (TCA), or mirtazapine, except that some patients were additionally treated with atypical antipsychotic drugs (olanzapine or quetiapine), lithium or lamotrigine to potentiate antidepressant therapy. The detailed characteristics of the study participant were presented in our previous report [30].

The mean age in the group of patients (49.36 ± 10.67) did not show significant differences from the control group (45.82 ± 12.43), (*p* = 0.064; Mann–Whitney *U* test). Nor there were any significant differences in terms of the gender distribution (*p* = 0.64; *χ* ^2^ test). The percentage of women in the group of patients was 75.4 %, and in the control group 72 %. There were no significant differences between the average serum concentration of copper in women and in men (*p* = 0.21; Mann–Whitney *U* test).

The mean scores in MADRS and HDRS among all the MDD patients (in depressive episode and remission together) were as follows: 17.64 ± 13.4 in MADRS and 13.02 ± 8.94 in HDRS. The mean MADRS and HDRS scores of patients in depressive group were 26.17 ± 9.74 and 18.83 ± 6.3, respectively. Among patients in remission mean MADRS scores were 4.25 ± 4.2, while HDRS 4.11 ± 3.14.

The analysis of variance indicated that there was no significant influence of the disease nor affiliation to healthy volunteer group on the obtained serum copper concentrations (*p* > 0.05; Kruskal–Wallis test). The mean copper levels in depression (0.81 ± 0.27 μg/ml) were lower than in the control group (0.91 ± 0.39 μg/ml). However, this difference was not statistically significant (*p* > 0.05). Similar lack of significance (*p* > 0.05) in copper concentration was observed between depressive patients and those in remission (1.01 ± 1.51 μg/ml) (Table 1).

**Table 1:** Serum copper concentration [μg/ml] in MDD patients, depending on the current phase of the disease (depression or remission), and healthy controls. Data are expressed as mean ± SD or Median [Upper/Lower quartile]

	Depression	Remission	Control
Cu concentration [μg/ml]	Mean ± SD		
	0.81 ± 0.27	1.01 ± 1.51	0.91 ± 0.39
	Median [upper/lower quartile]		
	0.83 [0.58/1.00]	0.85 [0.69/0.96]	0.82 [0.68/1.04]

Detailed analysis of the data obtained from patients with a various clinical picture of depressive episodes revealed that there were no statistically significant difference (*p* > 0.05; Mann–Whitney *U* test) in copper levels between patients with or without the following clinical features: atypical symptoms of depression, psychotic symptoms, melancholic syndrome, or drug resistance (Table 2).

**Table 2:** Comparison of copper serum concentrations [μg/ml; mean concentration, and median (upper/lower quantile)] in MDD patients with a various clinical picture of depressive episodes

Depression	Mean ± SD	Median [upper/lower quartile]	Mann–Whitney *U* test; *p*
With atypical features	0.70 ± 0.34	0.80 [0.46/0.93]	0.92
Without atypical features	0.93 ± 1.07	0.83 [0.68/0.99]	
With melancholic features	0.82 ± 0.28	0.81 [0.58/1.03]	0.63
Without melancholic features	0.93 ± 1.19	0.84 [0.58/0.98]	
With psychotic syndromes	0.73 ± 0.21	0.69 [0.56/0.90]	0.59
Without psychotic syndromes	0.89 ± 1.0	0.83 [0.64/1.0]	
Drug-resistant	0.71 ± 0.28	0.66 [0.46/0.84]	0.21

Another analysis also showed the lack of correlation between copper concentration in the depression or remission phase and age of the patients and several clinical features: duration of the disease; age at the onset of the disorder; duration of the current depressive episode or remission; average number of depressive episodes in last year; and number of total hospitalization in over the past year and in the lifetime. Only in the remission group significant correlation was observed between the serum copper concentration and the mean number of depressive episodes over the past year and duration of the remission (*r* = 0.30 and *r* = −0.41, respectively; *p* < 0.05).

The analysis of correlation between the copper levels and the severity of depression (as measured with the HDRS/MADRS total scores) showed no significance regardless of the subsample (Table 3).

**Table 3:** Correlations between serum copper concentration [μg/ml] and selected quantitative clinical features in depression and remission (Spearman correlation, *p < 0.05)

	Cu [μg/ml]		
	MDD (Total)	Depression	Remission
Age	−0.1	−0.08	−0.16
Age of disease onset	−0.021	−0.06	0.009
Number of episodes in the life	−0.012	0.1	0.18
The average annual number relapses in the last year	0.096	0.07	**0.30**
The disease duration	−0.057	0.0001	−0.16
Duration of the episode /remission	−0.177	−0.05	**−0.41**
Total MADRS score	0.001	0.013	0.03
Total HDRS score	−0.001	0.04	0.012

## Discussion

In this case–control study we found that the mean serum copper levels in patients with the depressive episode did not significantly differ from those obtained in healthy volunteers samples. Likewise, there were no significant differences between copper concentration in depressed patients with or without such clinical features as: atypical features of depression, drug resistance, presence of psychotic symptoms or melancholic syndrome. The obtained copper concentrations showed no correlation with many of the clinical features of the disease. However, in the remission group we found significant correlation between serum copper levels and number of episodes in last year or duration of the remission.

Until now, an analysis of copper concentration in the blood of the patients diagnosed with MDD was the subject of only a few case–control studies and all of them were conducted on a smaller groups of patients [6, 12–16]. Manser et al. [12] in a study of Karachi population observed elevated (by 22 %) plasma copper level in 31 (15 males, 16 females) depressed patients as compared to the 62 normal healthy individuals. Similar observations were also made later by Narang et al. [13]. In the study including 35 (21 males, 14 females) patients there were noted significantly higher (by 14 %) Cu levels in depressed people than in the healthy volunteers (*n* = 35). In turn, after recovery, plasma copper concentration were significantly decreased (by 15 %) [13]. Both of these studies seem to be in opposition to our results, which present no alterations in the level of copper in the course of depression. On the other hand, another study performed on a group of 31 depressed patients and 15 healthy volunteers by Maes et al. [6] indicating no change in copper concentration between the study groups. Additionally, Maes et al. reported no difference in patients meeting (or not) the criteria of melancholic syndrome or drug resistance and no significant correlation between copper levels and severity of depression, duration of depressive episode, age at the onset or total duration of the disorder [6]. Those results remain in line with our study. However, in the Maes et al.’s trial, there was a decline of copper levels in depressive patients following the antidepressant treatment, while we did not perform any corresponding analyses. Partly consistent with our findings are also the results obtained by Schlegel-Zawadzka et al. [15, 16]. Although, in the study, which enrolled 19 MDD patients and 16 healthy, was shown elevated levels of copper in depressed people. However, just like in our study, there was not any correlation between copper levels and severity of depression measured by HDRS, and moreover, effective antidepressant was without effect on this measure [15, 16]. Interesting observations, which in part are consistent with our results, have also been made in the cross-sectional analysis by Crayton and Walsh [11], the study to which 813 (485 women and 328 men) depressed patients and 54 (28 women and 26 men) control subjects between 30 and 60 years were selected; significant increase in the serum copper concentration in depressed women compared to the matched control was demonstrated. Additionally, it has been shown that women with a history of postpartum depression (PPD) exhibited significantly higher Cu level than those without PPD history. Simultaneously, no difference between the nondepressed and depressed men, and moreover, lower copper levels in normal men than in healthy women were observed. Unfortunately, although this transversal study was conducted on a large population, had a lot of limitations (e.g., absence of current formal diagnostic evaluations or small size of the control groups), which may undermine the potential usefulness of these results [11].

In line with our observations seem to be also the results of some animal models of depression (chronic severe stress, chronic mild stress and olfactory bulbectomy), that revealed no alterations in serum copper in relation to the control group [15].

Concluding, observed (not only) in our study, no significant differences in the serum copper concentration between the patients with the current episode of depression and healthy volunteers, between remission and healthy control group, nor the lack of correlation between the Cu level and severity of depression (or clinical features of population) may suggest that the role of this trace element in the pathophysiology of major depressive disorder and thereby its potential usefulness as a clinical marker of the disease seems to be relatively low.

On the other hand, the interpretation of our findings, apart from the strengths of the study (many possible intervening variables: age, sex, nicotine dependence, socio-demographic data and the drug state of the patients) should also take into account its restrictions: the lack of a prospective model to test the dynamics of the copper concentrations in the same patients, depending on the stage of the disease; substantial heterogeneity (with regard to the applied therapy) of the study groups and the relatively small number of subgroups presenting specified clinical features. It is also important to mention about significance of zinc/copper ratio, which is crucial for proper functioning of human organism [8]. Some researcher suggest that zinc-copper imbalance can lead to the development of many diseases, especially psychiatric disorders (e.g., depression, postpartum depression, schizophrenia, autism spectrum disorders) and that monitoring of Zn/Cu ratio may be clinically a more sensitive and reliable marker than measuring of each element separately [see 8 for review].

Regardless of this fact, no doubt, this study represents a significant source of knowledge on this issue; however, in view of many limitations and an extremely small number of published reports (and ambiguity of results), our observations should be confirmed by subsequent studies conducted on an even larger (and also other) human population and allowing for analysis of the impact of applied pharmacotherapy and other clinical factors on the copper concentration.

## Limitations

The main limitations of the presented study are the following: lack of a prospective model to test the dynamics of changes in copper levels in individual patients; considerable heterogeneity of the study groups in terms of treatment; and the small number of subgroups presenting specified clinical features.
